# Transbilayer phospholipids molecular imaging

**DOI:** 10.1186/2191-219X-1-17

**Published:** 2011-08-22

**Authors:** Tarik Z Belhocine, Frank S Prato

**Affiliations:** 1 Department of Medical Imaging, The University of Western Ontario, London, ON, Canada; 2 Departments of Medical BioPhysics and Physics, Lawson Health Research Institute, The University of Western Ontario, London, ON, Canada

**Keywords:** phospholipids, molecular imaging, PET-CT, SPECT-CT

## Abstract

Nuclear medicine has become a key part of molecular imaging. In the present review article, we focus on the transbilayer phospholipids as exquisite targets for radiolabelled probes in molecular imaging. Asymmetry of phospholipid distribution is a characteristic of mammalian cell membranes. Phosphatidylcholine and sphyngomyelin cholinophospholipids are primarily located within the external leaflet of the cell membrane. Phosphatidylserine and phosphatidylethanolamine aminophospholipids, and also phosphatidylinositol are primarily located within the internal leaflet of the cell membrane. New radiolabelled tracers have been designed in preclinical and clinical research for PET-CT and SPECT-CT molecular imaging of transbilayer phospholipids.

## Introduction

In this beginning of the twenty-first century, *personalized molecular medicine *is the objective of molecular diagnosis, molecular imaging, and molecular therapy [1,2]. Molecular imaging (MI) includes a number of morphological imaging techniques (i.e. ultrasound, computed tomography and magnetic resonance imaging), and optical imaging techniques (i.e. bioluminescence and fluorescence imaging) [3,4]. In addition, nuclear medicine with radiolabelled probes has become a key part of MI, especially with ^18^F-fluorodeoxyglucose (^18^F-FDG) metabolic imaging [5]. New radiolabelled tracers have been designed for positron emission tomography-computed tomography (PET-CT) and single-photon emission computed tomography-computed tomography (SPECT-CT) molecular imaging [6]. In this review article, we focus on the transbilayer phospholipids as exquisite targets for radiolabelled probes in molecular imaging.

### Molecular imaging

There is no universally accepted definition of molecular imaging [5,7]. In 2000, the Society of Molecular Imaging http://www.molecularimaging.org/ defined *molecular imaging *as: 'the characterization and measurement of biological processes in living animals at the cellular and molecular level'. In 2005, the European Society for Molecular Imaging http://www.e-smi.eu formulated a definition of molecular imaging as: 'the characterisation of the dynamics of the molecular processes in the living organisms in vivo. In vivo molecular imaging is a science combining molecular biology, cellular biology and physiology with imaging in living subjects'. In 2006, the Federation of Asian Societies for Molecular Imaging (FASMI: http://fasmi.org/) defined molecular imaging as: ' the characterization and measurement of biological processes in living animals at the cellular and molecular level by means of non-invasive (or minimally invasive) imaging'. In 2007, the Society of Nuclear Medicine Molecular Imaging Center of Excellence http://interactive.snm.org/ definitions task force approved this definition of molecular imaging as: 'the visualization, characterization, and measurement of biological processes at the molecular and cellular levels in humans and other living systems' [8]. A MI *probe *is a molecule used in molecular imaging to deliver a tracer to a specific organ or tissue. A probe typically consists of a ligand containing or linked to a signalling label. The label provides the signal (i.e. electromagnetic wave, light and radiation) that can be picked up by a detector, and the ligand carries the tracer to the site of interest [9]. A MI *target *used in molecular imaging is a molecule or structure in the body to which binds a probe delivered to a specific organ or tissue. The target may be a peptide, or a glucide, or a lipid; in many cases, the target is a protein [10,11]. Molecular imaging may be a single disease/gene or a general disease/biologic function control point for targeting [12].

Molecular imaging with transbilayer phospholipid targets may be performed with radiolabelled probes such as radiolabelled annexin V or C2A synaptotagmin domain I or beta 2 glycoprotein I, radiolabelled duramycin, radiolabelled hypericin, radiolabelled lactadherin, radiolabelled choline or fluorocholine, radiolabelled diacylglycerols, radiolabelled sphyngomyelin for visualization, characterization and measurement of key biological functions (i.e. apoptosis, necrosis, thrombosis, vasculature endothelium, choline metabolism, myocardial and neuronal phosphoinositide turnover) or for assessing specific diseases (i.e. cancers, immune diseases, inflammatory diseases, infectious diseases, cardiac diseases and neurological diseases).

### Membrane bilayer

The membrane bilayer is composed of 40% lipids and glycolipids, and 60% integral proteins and glycoproteins [13]. The lipids in the membrane bilayer are composed of phospholipids (75% to 88%), glycosphyngolipids (2% to 5%) and cholesterol (10% to 20%) [13]. The phospholipids include phosphatidylcholine (45% to 55%), phosphatidylethanolamine (15% to 25%), phosphatidylinositol (10% to 15%), phosphatidylserine (2% to 10%), phosphatidic acid (1% to 2%), sphyngomyelin (5% to 10%) and cardiolipin (2% to 5%). Liposomes are artificial lipid *vesicles *encapsulating drugs (e.g. chemotherapy drugs, antibiotics, fungicides), enzymes, biological material (e.g. antigens, antibodies) and tracers (e.g. radiolabelled products, contrast agents) [14]. Liposomes are nanoparticles with a diameter < 100 nm characterised by the composition of lipids, the number of membrane bilayers, and the surface charges [7]. The material encapsulated is either dissolved in an aqueous phase or in a lipid phase. Radioactive phospholipid liposomes have been designed for molecular imaging [15].

### Phospholipid bilayer

**Figure 1:**
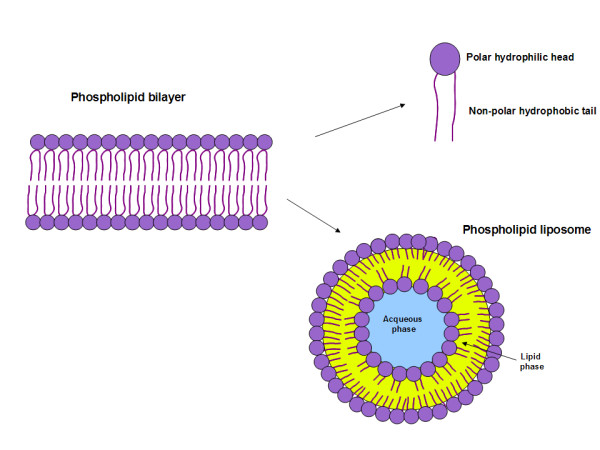
**Phospholipid bilayer composed of hydrophobic non-polar tails and hydrophilic polar heads**. Liposomes are artificial phospholipid vesicles encapsulating materials either in an aqueous phase or in a lipid phase.

**Figure 2:**
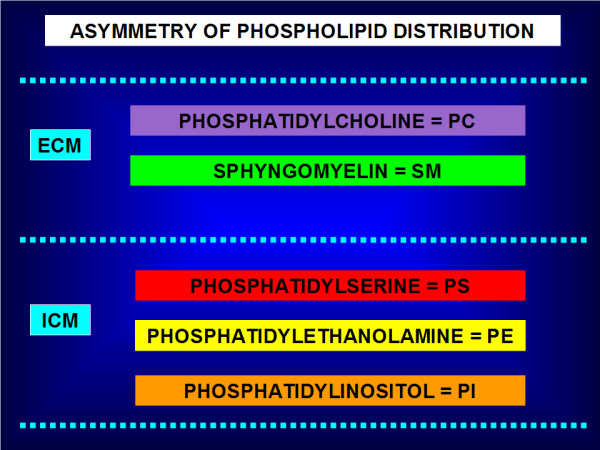
**Asymmetry of phospholipid distribution**. Phosphatidylcholine (PC) and sphyngomyelin (SM) cholinophospholipids are primarily located in the external leaflet of the cell membrane (ECM). Phosphatidylserine (PS) and phosphatidylethanolamine (PE) aminophospholipids as well as phosphatidylinositol (PI) phospholipid are primarily located in the internal leaflet of the cell membrane (ICM). In mammalians, the asymmetry of phospholipid distribution is maintained by the flip/flop movement of phospholipids. PS and PE are maintained to the inner leaflet of the membrane bilayer by the Mg^2+^-ATPase (i.e. aminophospholipid translocase), and the ATP-dependent aminophospholipid-specific flippase and cholinophospholipid-specific floppase (i.e. multidrug resistance proteins). In pathophysiological conditions, PS and PE are shuttled to the outer leaflet after inactivation of the aminophospholipid translocase or flippase, and activation of the Ca^2+^-dependent ATP-independent scramblase.

In 1972, Singer and Nicolson developed *the fluid mosaic model *to explain the composition of the cell membrane bilayer with randomly oriented globular proteins and lipids [16]. According to this thermodynamic model, the phospholipid membrane bilayer is composed of hydrophilic heads and hydrophobic tails. The polar hydrophilic heads are in contact with water (see Figure 1). Recent refinements of the Singer-Nicolson mosaic model suggest a structured dynamic organisation of the membrane bilayer with genetically predefined non-randomly oriented globular proteins into the lipid matrix [17,18]. In mammalians, the *asymmetry *of phospholipid distribution is a characteristic of cell membranes (see Figure 2). Phosphatidylcholine (PC) and sphyngomyelin (SM) cholinophospholipids are primarily located in the extracellular membrane. Phosphatidylethanolamine (PE) and phosphatidylserine (PS) aminophospholipids as well as phosphatidylinositol (PI) phospholipid are primarily located in the intracellular membrane [19]. Two ATP-dependent translocase enzymes (i.e. cholinophospholipid-specific translocase or floppase, and aminophospholipid-specific translocase or flippase) and a Mg^2+^-ATPase (i.e. aminophospholipid translocase) maintain the aminophospholipids in the inner leaflet of the membrane bilayer. PE and PS are externalised to the outer leaflet of the cell membrane after inactivation of aminophospholipid translocase or flippase and activation of scramblase, another Ca^2+^-dependent ATP-independent enzyme [19,20].

### Phospholipid targets

Phospholipids are composed of a phosphatidyl tail including a free fatty acid, glycerol and phosphate [21]. In the phospholipid polar head, choline, serine, ethanolamine and inositol bind to the phosphatidyl non-polar tail. Sphingomyelin is the only one cell membrane phospholipid not derived from a glycerol but from an aminoalcohol sphingosine; SM is composed of a core ceramide (i.e. sphingosine and free fatty acid tail) and contains a polar head group composed of phosphocholine or phosphoethanolamine [22].

**Figure 3:**
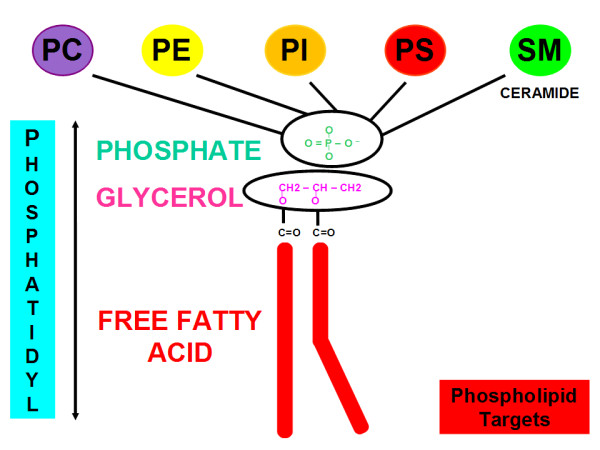
**Phospholipid targets are composed of a phosphatidyl tail including free fatty acid, glycerol, and phosphate**. In the phospholipid polar head, choline, serine, ethanolamine, and inositol bind to the phosphatidyl non-polar tail. Sphingomyelin is the only one cell membrane phospholipid not derived from a glycerol but from an aminoalcohol sphingosine; SM is composed of a core ceramide (i.e. sphingosine and free fatty acid tail) and contains a polar head group composed of phosphocholine or phosphoethanolamine.

In preclinical and clinical research, phospholipids are used as targets for molecular imaging with radiolabelled probes as illustrated in Figure 3[23,24].

### PS target

PS is an aminophospholipid located in the inner leaflet of the cell membrane. In pathophysiological conditions including apoptosis, thrombosis and tumour vasculature, PS is externalised from the inner leaflet to the outer leaflet of the cell membrane [25]. In necrosis, PS is exposed in the inner leaflet of damaged cells. For SPECT-CT and PET-CT imaging, radiolabelled probes have been designed to target exposed PS with or without the breakdown of cell membrane asymmetry.

**Figure 4:**
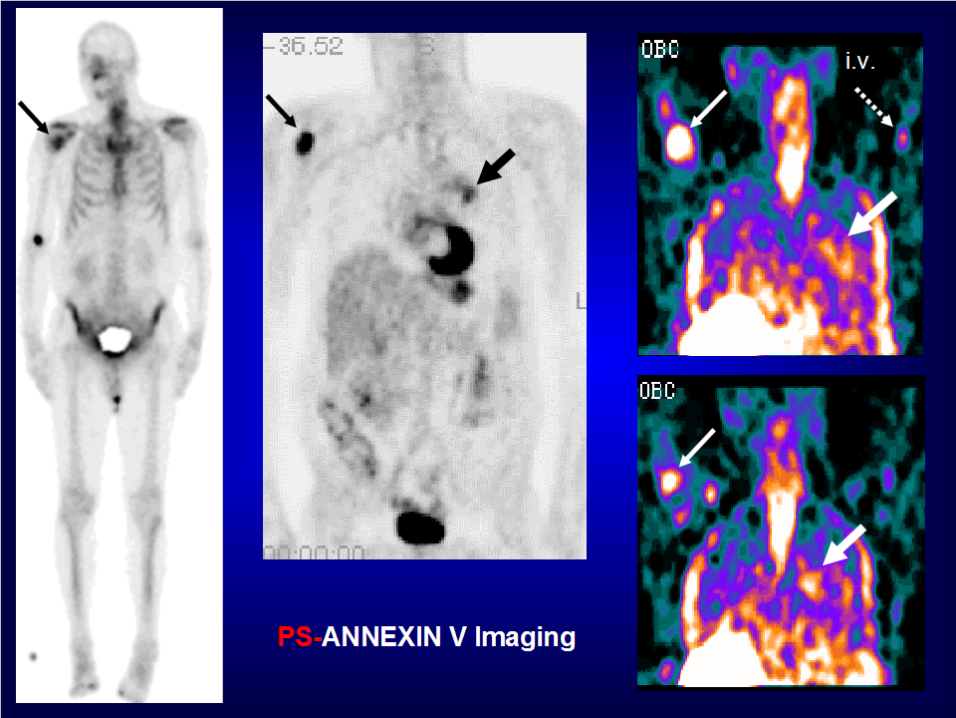
**PS-annexin V imaging (North American Scientific 2020 - Phase II-III - Clinical Trial - Antwerp 2003)**. A patient with a left para-hilar NSCLC (large black arrow) who presented with a bone metastasis in the right upper humerus (fine black arrow) on bone scan and ^18^FDG PET. ^99m^Tc-HYNIC-annexin V imaging showed tracer uptake in the primary tumour (large white arrow) and the bone metastasis (fine white arrow) as soon as 24 h after the first course of chemotherapy. i.v., intravenous injection (dashed arrow).

**Figure 5:**
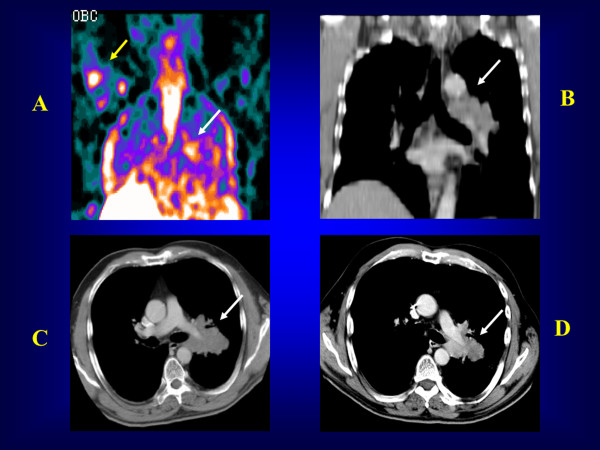
**PS-annexin V imaging (North American Scientific 2020 - Phase II-III Clinical Trial - Antwerp 2003)**. A patient with a left para-hilar NSCLC on pre-chemotherapy CT (upper right: simple white arrow) who presented with ^99m^Tc-HYNIC-annexin V uptake post-chemotherapy in the primary lung tumour (upper left: simple white arrow) and the right upper humerus bone metastasis (upper left: yellow arrow). After the first course of chemotherapy, diagnostic CTs showed a decrease in the primary lung tumour greatest diameter, which was consistent with an apoptotic tumour response to chemotherapy (lower left and lower right: simple white arrows). Upper left (**A**): post-chemotherapy ^99m^Tc-HYNIC-annexin V scan; upper right (**B**) and lower left (**C**): pre-chemotherapy diagnostic CTs; lower right (D): post-chemotherapy diagnostic CT.

***Annexin V***, a 36-kDa serum protein first isolated from human placenta, has been radiolabelled with ^99m^Tc, ^111^In, ^67^Ga, ^123^I, ^124^I, ^11^C, ^64^Cu, ^68^Ga, ^94m^Tc and ^18^F for imaging of apoptosis or necrosis, and thrombosis [26-29]. Annexin V binds specifically to PS in presence of Ca^2+ ^ions. Spontaneous and induced apoptotic changes may be assessed qualitatively and quantitatively with radiolabelled annexin V-targeted to PS [30,31]. In preclinical research, ^99m^Tc-HYNIC-annexin V imaging has been used to evaluate cardiovascular models (e.g. heart transplant rejection, myocarditis and mural thrombus), oncology models (e.g. cyclophosphamide induced intramedullary and splenic apoptosis in lymphoma), neurology models (e.g. neonatal brain ischemia), inflammatory/infectious models (e.g. fulminant hepatitis, subacute and acute infection) and immune models (e.g. rheumatoid arthritis treated by corticosteroids). In clinical research, ^99m^Tc-HYNIC-annexin V imaging using a recombinant human form of annexin V has been used to assess myocardial infarction, heart transplant rejection, atherosclerosis, ischemic pre-conditioning and chemotherapy/radiation therapy-induced apoptosis in head and neck cancers, lymphomas, lung cancers, breast cancers, leukaemia and soft tissue sarcomas. ^99m^Tc-HYNIC-annexin V imaging has also been used in patients with dementia and stroke, and in patients with rheumatoid arthritis and Crohn's disease for evaluation of early apoptotic response to Infliximab^® ^therapy (i.e. anti-TNFα monoclonal antibody) [24,26]. Figures 4 and 5 illustrate PS targeting ^99m^Tc-HYNIC-annexin V molecular imaging probe in a patient with a non-small cell lung cancer (NSCLC). PS targeting ^99m^Tc-annexin V-128 with an N-terminal site specific for endogenous binding of technetium-99m has been designed in order to improve the sensitivity of detection of apoptosis or necrosis (i.e. twofold higher than that of ^99m^Tc-HYNIC-annexin V) [32].

***C2A domain of synaptotagmin I ***(C2A) is a 12-kDa protein predominantly located within the synaptic vesicles binding to PS. It has been fused with glutathione-s-transferase (GST) and radiolabelled with ^99m^Tc for imaging of apoptosis or necrosis [33]. In a rat and pig model of acute myocardial infarction, increased ^99m^Tc-C2A-GST uptake was seen in the myocardial infarct area at risk, and was associated with wall dysfunction [34-36]. In a mouse model of NSCLC, increased ^99m^Tc-C2A-GST tumour uptake was noted after paclitaxel chemotherapy-induced apoptosis [37]. Also, C2A-GST has been easily labelled with ^18^F for early imaging of apoptosis after chemotherapy [38]. In a rabbit model of lung cancer, increased ^18^F-C2A-GST tumour uptake was detected 72 h after paclitaxel chemotherapy with increased apoptotic index and caspase-3 activity. Recently, the C2A domain of synaptotagmin I has been labelled with a [^99m^Tc(CO)_3_^+^] core by using an efficient C-terminal site-specific radiolabelling method for the imaging of cell death [39].

PS is not exposed in normal endothelium, but increased exposure of PS is seen on the tumour endothelium vasculature [40,41]. ***Bavituximab***, a chimeric monoclonal antibody (MW = 145.3 kDa) binding to the beta-2 glycoprotein I domain of PS, has been radiolabelled with the β + emitter arsenicum-74 (^74^As, *T*_1/2 _= 17.8 days) for tumour vasculature PET imaging, and with the β-,γ emitter ^77^As (*T*_1/2 _= 38.8 h) for SPECT imaging and potential tumour vasculature endothelium therapy [42-44]. Increased ^74^As-bavituximab uptake was seen in a rat model of prostate cancer with the highest tumour-to-background activity ratio at 72 h post-i.v injection (at 72 h p.i., tumour-to-liver ratio = 22, and tumour-to-muscle ratio = 470). Using autoradiography and immunohistochemical studies, ^74^As-bavituximab was found to specifically bind to the tumour endothelium vasculature. Bavituximab radiolabelled with ^77^As or ^76^As (β-, *T*_1/2 _= 26.3 h) may be used for dosimetry and immunotherapy.

***Hypericin***, a non-porphyrin necrosis agent (MW = 504 Da) extracted from St John's wort, has been labelled with ^64^Cu (β+, *T*_1/2 _= 12.7 h) for PET imaging of necrosis. In a female mouse model of BT474 breast xenograft tumour, ^64^Cu-bis-DOTA-hypericin demonstrated increased uptake at 24 h post-i.v. injection in necrotic injured tissues treated with near infrared photothermal ablation therapy. PET distribution also showed higher uptake in the liver and the kidneys. Bis-DOTA-hypericin had a selective binding affinity for PS and PE phospholipids [45]. In necrosis, PS and PE are exposed to ^64^Cu-labelled hypericin probe in the inner leaflet of damaged cell membrane. Hypericin derivatives are efficient and yield reproducible results when radiolabelled with ^123^I (i.e. mono-^123^I-iodohypericin and mono-^123^I-iodohypericin monocarboxylic acid). They have been also used in preclinical models of liver necrosis and myocardial infarction as well as in clinical correlates of these pathophysiologic states [46,47]. In a preclinical rat model of liver rhabdomyosarcoma, ^131^I-labelled hypericin was successfully used in theragnostics with a vascular disrupting agent (i.e. combretastatin A4 phosphate or CA4P); high radiolabelling efficiency was noted with minimum deiodination. ^131^I-hypericin uptake colocalised tumour necrosis within 24 h post-i.v. injection on co-registered planar γ scintigraphy with CT, MRI, histology and autoradiography [48]. Hypericin has also been labelled with ^99m^Tc (i.e. ^99m^Tc-hypericin or ^99m^Tc-mercaptoacetyldiglycyl-1,5-diaminopentylene hypericin-carboxamide) for visualisation of necrotic tissues in rats with reperfused liver infarct [49]. ^99m^Tc-hypericin, however, was found to be not suitable for imaging of necrosis compared to ^123^I-hypericin derivatives [50]. Although the mechanism of target uptake is unknown, it may be hypothesised that the PS and PE phospholipid targets are involved for ^123^I-, ^131^I- and ^99m^Tc-labelled hypericin derivatives, but this is still to be definitely proven.

***Lactadherin***, a glycoprotein secreted by mammary epithelium, epididymal epithelium, vascular cells and activated macrophages, has been shown to bind in a Ca2+-independent manner and specifically to the C2 domain of PS [51-53]. *In vitro*, FITC-labelled bovine lactadherin has been used for early detection of apoptosis in leukaemia cell lines treated by etoposide, and also in HeLa cervical cancer lines treated by staurosporine [54,55]. HYNIC-lactadherin has also been successfully labelled with ^99m^Tc (i.e. ^99m^Tc-HYNIC-lactadherin). *In vivo*, in a mouse model, ^99m^Tc-HYNIC-lactadherin had a lower uptake in the kidneys compared to ^99m^Tc-HYNIC annexin V. However, this new PS-targeting probe had a higher uptake in the liver, which is a disadvantage for imaging of liver and myocardial apoptosis [56].

***PSBP-6 ***or PS-binding peptide-6, a synthetic 14 amino acid sequence (MW = 1.623 kDa; Kd = 100 nM) where Gln is replaced by Ala screened through a peptide library, showed the highest relative binding affinity and stability; a single amino acid chelator (SAAC) was introduced at the N-terminus position of PSBP-6 to form a stable complex with ^99m^Tc (i.e. SAAC-^99m^Tc-PSBP-6), and to improve the labelling efficiency. Unlike annexin V, SAAC-PSBP-6 binding to PS is Ca^2+ ^independent [57]. In DLD1 human colon carcinoma cells pretreated with increased TRAIL doses and also in murine melanoma B16/F10 cells treated with poly(L-glutamic acid) paclitaxel (PG-TXL), SAAC-(^99m^Tc)-PSBP-6 showed selective binding to apoptotic cells *in vitro*; confirmation was obtained with TUNEL staining and autoradiography. In treated and untreated nude mice, biodistribution data showed increased SAAC-(^99m^Tc)-PSBP-6 uptake in the liver and the kidney *in vivo*. Preclinical studies are required to assess the sensitivity and specificity of this new PS binding molecular imaging probe. Table 1 summarises radiolabelled PS-based molecular imaging probes.

**Table 1:** Phosphatidylserine-based radiolabelled molecular imaging probes

Molecular target	Molecular probes	AA	MW (kDa)	Affinity (M)	Biological functions	Isotopes	Imaging techniques
PS ^a^	annexin V	319	36	10^-9^	apoptosis, necrosis, thrombosis	^99m^Tc, ^123^I, ^111^In, ^67^Ga, ^18^F, ^68^Ga, ^124^I, ^64^Cu, ^94m^Tc	SPECT(CT), PET(CT)
PS	C2A domain of synaptotagmin I	128	12	10^-9^	apoptosis, necrosis	^99m^Tc	SPECT(CT)
						^18^F	PET(CT)
PS ^b^	bavituximab chimeric antibody	-	145.3	10^-9^	tumour endothelium vasculature imaging	^74^As ^c^	PET(CT)
					tumour endothelium vasculature therapy	^77^As ^d^	SPECT(CT)
PS	hypericin	-	0.504	-	necrosis	^99m^Tc, ^123^I, ^131^I ^64^Cu ^e^	SPECT(CT) PET(CT)
PS	lactadherin	364	47 - 50	10^-9^	apoptosis	^99m^Tc ^f^	SPECT(CT)
PS	PSBP-6	14	1.623	10^-9^	apoptosis, necrosis	^99m^Tc ^g^	SPECT(CT)

^a ^Various linkers have been used for the radiolabelling of annexin V [26]. HYNIC is the most used bifunctional chelating agent in preclinical and clinical research. ^b ^β2 glycoprotein I domain of phosphatidylserine. ^c 74^As, β + emitter (*T*_1/2 _= 17.8 days). ^d 77^As, β- emitter (*T*_1/2 _= 1.6 days). ^e ^Bis-DOTA has been used as a bifunctional chelating agent for radiolabelling of hypericin. ^f ^HYNIC has been used as a bifunctional chelating agent for radiolabelling of lactadherin. ^g ^SAAC has been used as a single amino acid chelator for radiolabelling of PSBP-6. AA, amino acids; MW, molecular weight; PET(CT), positron emission tomography (computed tomography); PS, phosphatidylserine; PSBP-6, PS-binding peptide-6; SPECT(CT), single-photon emission computed tomography (computed tomography).

### PC target

Choline is a main component of biomembranes targeted by the choline kinase enzyme, and is phosphorylated to intracellular phosphocholine and extracellular phosphatidylcholine [58]. Choline has been labelled with ^11^C (i.e. ^11^C-choline) and more recently with ^18^F (i.e. ^18^F-fluorocholine) for PET-CT imaging [59,60]. With a longer physical half-life, ^18^F-fluorocholine (^18^F-FCH; ^18^F, *T*_1/2 _= 109 min) is more suitable than ^11^C-choline (^11^C, *T*_1/2 _= 20 min) for PET-CT imaging. In human subjects, ^18^F-FCH showed a fast blood pool clearance with a peak ≤ 5 min post-i.v. injection, a fast tissue uptake and a predominantly renal excretion. Additionally, ^18^F-FCH biodistribution changes very slowly later than 10 min after i.v. injection [61].

***^18^F-fluorocholine ***(^18^F-FCH) and ***^11^C-choline ***are incorporated into the membrane phospholipids, and are predominantly used for prostate cancer imaging and brain tumour imaging [62,63]. In prostate cancer patients, ^18^F-FCH is a promising tracer for detection of primary prostate cancer, staging of lymph node and bone metastases, and detection of recurrence after definitive therapy [62-64]. In brain tumours, this agent may be useful to detect glioblastoma multiforme, to distinguish high-grade gliomas with a characteristic peri-tumoural uptake from metastases and benign lesions, to guide a stereotactic biopsy, and also to differentiate post-radiation necrosis from recurrence [65-68]. Also, ^11^C-choline and ^18^F-FCH have been used for hepatocellular carcinoma (HCC) imaging [69-71]. In a woodchuck model, ^11^C-choline sensitivity for detection of well-differentiated HCCs was higher than that of ^18^F-FDG, and in 12 patients with moderately differentiated HCCs, the ^11^C-choline detection rate was better than that of ^18^F-FDG. ^18^F-FCH imaging has also successfully been performed for detection of well-differentiated HCC in patients with liver nodules or cirrhosis or chronic liver disease, and for detection of recurrences from HCC [72,73].

Under normal glucose conditions, acetate free fatty acid is metabolised into acetyl-CoA by acetyl-CoA synthetase in both the cytosol and mitochondria for synthesis of cholesterol and fatty acids [74]. In tumours, acetate is metabolised into fatty acids by fatty acid synthetase, which is overexpressed in cancer cells [75]. Acetate is mainly incorporated in the PC microdomains that play a major role for growth and metastasis [76]. Acetoacetate ketone body is metabolised to acetoacetyl-CoA and to acetyl-CoA, and then incorporated into the metabolic pathway for lipid synthesis [77]. Like acetate, acetoacetate could be incorporated into the PC membrane. Acetate and acetoacetate have been labelled with ^11^C (i.e. ^11^C-acetate and ^11^C-acetoacetate) [74,77]. Acetate has also been labelled with ^18^F (i.e. ^18^F-fluoroacetate) [78]. ***^11^C-acetate ***has a rapid blood clearance with a high accumulation in liver and myocardium. In normal physiology, ***^18^F-fluoroacetate ***is not a functional analogue of ^11^C-acetate; ^18^F-fluoroacetate has a longer blood half-life, a rapid liver clearance and low myocardial uptake, extensive excretion into bile and urine, and suffers from skeletal defluorination resulting in skeletal uptake of the formed ^18^F-fluoride [79]. In preclinical and clinical research, ^11^C-acetate PET imaging has been successfully used for tumour imaging in cancers of the prostate, kidney, pancreas, as well as gliomas, meningiomas and HCC [80,81]. In preclinical research, ^18^F-fluoroacetate has been used for imaging of gliosis in glioblastoma, stroke, and ischemia-hypoxia associated with neuroinflammation [82]. In CWR22 tumour-bearing male *nu/nu *mice, ^18^F-fluoroacetate has been successfully used for prostate cancer imaging with a higher tumour-to-prostate activity ratio compared to ^11^C-acetate at 30 min and 2 h post-i.v. injection. In a patient with prostate cancer, ^18^F-fluoroacetate was also able to detect several but not all bone metastases [83,84]. In other preclinical research models of breast and prostate cancers, ***^11^C-acetoacetate ***uptake tended to be higher than that of ^11^C-acetate [85]. Assessment of brain ketone metabolism in rats indicated a 7 to 8 fold increase in ^11^C-acetoacetate under ketone dietary treatment [86,87]. Table 2 summarises the radiolabelled PC-based molecular imaging probes.

**Table 2:** Phosphatidylcholine-based radiolabelled molecular imaging probes

Molecular target	Molecular probes	Biological functions	Isotopes	Imaging techniques
PC	choline	choline metabolism	^11^C	PET(CT)
PC	fluorocholine	choline metabolism	^18^F	PET(CT)
PC	acetate	acetate metabolism	^11^C	PET(CT)
PC	fluoroacetate	acetate metabolism	^18^F	PET(CT)
PC	acetoacetate	acetoacetate metabolism	^11^C	PET(CT)

PC, phosphatidylcholine; PET(CT), positron emission tomography (computed tomography).

### PE target

PE is primarily located in the inner leaflet of the membrane bilayer [88]. Like PS, this major aminophospholipid is externalised from the inner leaflet to the outer leaflet of the cell membrane during apoptosis and tumour vascular endothelium [89,90]. In necrosis, PE is exposed in the inner leaflet of disintegrated cell membrane.

**Figure 6:**
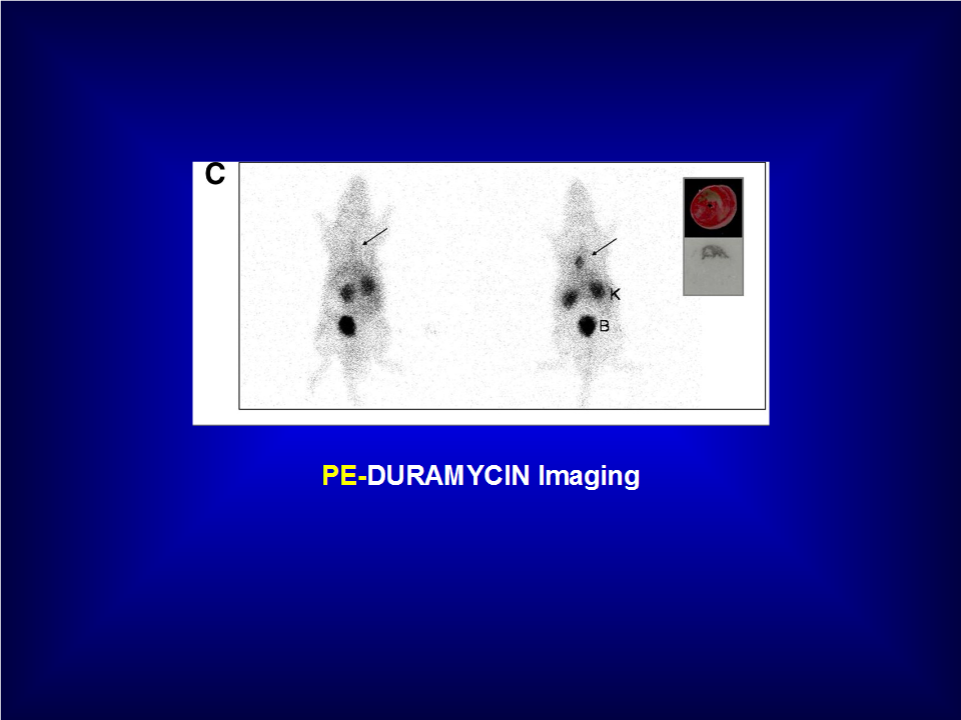
**PE-duramycin imaging**. Increased ^99m^Tc-Duramycin uptake is seen in acute myocardial infarction of the rat at 120 min post-i.v. injection with excellent infarct-to-non infarct activity ratio (right). Autoradiography confirmed radioactivity uptake within the myocardial infarction. No tracer uptake is seen in the healthy rat (left). B, bladder; K, kidney. Courtesy of Pr. Zhao M (Department of Biophysics, Medical College of Wisconsin, Milwaukee, WI, USA). Previously published in the *J Nucl Med *2008, 49(8):1345-52.

***Duramycin***, a 2-kDa small protein produced by *Streptovercillium cinnamoneus*, has been designed in order to target PE for imaging of apoptosis or necrosis [91]. ^99m^Tc-HYNIC-duramycin has been successfully used in a rat model of ischemia-reperfusion for imaging of myocardial infarction cell death [92]. ^99m^Tc-duramycin is highly stable *in vivo*, and has favourable pharmacokinetics for early imaging of acute cardiac cell death at 10 min post-i.v. injection with a fast blood clearance, a low liver uptake and a high uptake in apoptotic and necrotic myocardium. Figure 6 illustrates the PE targeting ^99m^Tc-duramycin molecular imaging probe in a rat model of myocardial infarction with correlation by autoradiography.

In addition to this, radiolabelled ethanolamine (i.e. ethanolamine labelled with ^11^C or ^18^F) is a potential new probe in oncology PET-CT imaging for assessment of tumour proliferation [93]. ***^14^C-ethanolamine ***has been used in a variety of tumour cell types (i.e. melanoma, prostate cancer, glioblastoma multiforme, diffuse large B-cell lymphoma, colorectal adenocarcinoma). ^14^C-ethanolamine is incorporated into PE and has a two- to sevenfold significantly better uptake into tumour cell types than ^14^C-choline. In an *in vitro *model of cultured prostate cancer cells, ^14^C-ethanolamine and ***^14^C-N, N'-dimethyl-ethanolamine ***uptake was two- to fourfold better in androgen-dependent and proliferating PC3 cells compared to androgen-independent and growth-arrested LnCap cells.

***Hypericin***, a PE- and PS-targeting probe, has been radiolabelled with ^64^Cu (i.e. ^64^Cu-bis-DOTA-hypericin), ^123^I (i.e. mono-^123^I-iodohypericin and mono-^123^I-iodohypericin monocarboxylic acid), ^131^I (i.e. ^131^I-hypericin) and ^99m^Tc (i.e. ^99m^Tc-hypericin) for imaging of necrosis in preclinical and clinical research as described above in the PS target section [45-50]. Table 3 summarises the PE-based radiolabelled molecular probes.

**Table 3:** Phosphatidylethanolamine-based radiolabelled molecular imaging probes

Molecular target	Molecular probes	AA	MW (kDa)	Affinity (M)	Biological functions	Isotopes	Imaging techniques
PE	duramycin	19	2	10^-9^	apoptosis necrosis	^99m^Tc ^a^	SPECT(CT)
PE	ethanolamine N'N-dimethyl ethanolamine	-	-	-	ethanolamine metabolism (tumour proliferation)	^14^C ^b^	PET(CT)
PE ^c^	hypericin	-	0.504	-	necrosis	^123^I, ^131^I ^99m^Tc	SPECT(CT)
						^64^Cu ^d^	PET(CT)

^a ^HYNIC has been used as a bifunctional chelating agent for the radiolabelling of duramycin. ^b ^ethanolamine or N,N'-dimethylethanolamine labelled with ^14^C in preclinical *in vitro *research may be labelled with ^11^C or ^18^F for PET(CT) *in vivo *imaging. ^c 64^Cu-bis-DOTA-hypericin has a selective binding affinity for PS and PE (ref.45); it may be hypothesised that ^123^I-, ^131^I- and ^99m^Tc-labelled hypericin derivatives bind to PS and PE. ^d ^bis-DOTA has been used as a bifunctional chelating agent for the radiolabelling of hypericin. AA, amino acids; MW, molecular weight; PE, phosphatidylethanolamine; PET(CT), positron emission tomography (computed tomography); SPECT(CT), single-photon emission computed tomography (computed tomography).

### PI target

PI phospholipid is located in the inner leaflet of the membrane bilayer [94]. 1,2 Diacylglycerol (DAG) is metabolised to intermediate phosphoinositide metabolites (i.e. PA, PIP, PIP_2_, PIP_3_) including phosphatidylinositol (PI) [95]. DAG activates the intracellular protein kinase C (PKC) transduction signalling pathway, which is involved in higher cortical functions (e.g. memory, learning) and cardiac functions (e.g. hypertrophic growth, ventricular remodelling).

***Intact ^11^C-inositol ***(i.e. non-acetylated inositol) has been suggested as a diagnostic agent in PET imaging for evaluation of PI brain metabolism and its role as second messenger [96]. In a rat and monkey model, 1-[1-^11^C]-butyryl-2-palmitoyl-*rac*-glycerol or ***^11^C-DAG ***has been designed as a PI-targeting probe in PET brain imaging to visualise neuronal PI turnover [97,98]. ^11^C-DAG levels are low in the brain, and thus it may be appropriately used to visualise PI metabolism in the central nervous system. Dynamic PET imaging showed increased ^11^C-DAG uptake in the first 15 min with an equilibrium at 16 min in pre-stimulation and post-stimulation conditions, which is suggestive of a *membrane trapping *mechanism. ^11^C-DAG uptake increased 20% to 30% after arecoline stimulation (i.e. acetylcholine muscarinic receptor stimulation), which suggests that ^11^C-DAG is a tracer of the transduction signalling PI-mediated pathway. In resting conditions, ^11^C-DAG uptake was observed in the visual association area, and increased in the whole brain and the occipital areas after arecoline stimulation. In C6 glioma cells implanted in the rat brain, ^11^C-DAG was rapidly incorporated in the PI turnover within 5 min post-i.v injection; in a patient with astrocytoma grade III, ^11^C-DAG was gradually incorporated in the leading PI turnover and the PE-PC secondary pool with an equilibrium period at 32-40 min post-iv. injection [99]. In patients with Alzheimer's disease and ischemic stroke, ^11^C-DAG evaluated the PI cortical function and neural viability, respectively. In eight patients with Alzheimer's disease, ^18^F-FDG PET imaging showed bilateral hypometabolism in the parieto-temporal association areas, while ^11^C-DAG imaging showed spotty uptake in the frontal lobes of the brain suggestive of compensatory plastic process in non-damaged neural circuits to degenerative cognitive impairment [100]. In five patients with cerebral infarction, dynamic PET brain imaging showed decreased ^11^C-DAG uptake in comparison to normal cortex [101]. ^11^C-DAG pharmacokinetics demonstrated rapid decrease in the plasma with a peak at 40 s post-i.v. injection, and gradual increase in the brain to reach a plateau at 15 to 20 min post-i.v. injection. Reflecting neural signal transduction activity, the incorporation constant of ^11^C-DAG (k*DAG) was best correlated with the cerebral metabolic rate of O_2 _(CMRO_2_). Maintained PI metabolism suggested preservation of neural viability in the peri-infarct area of the ischemic stroke. Patients with subacute local brain injury, either ischemic stroke or brain tumour, also exhibited ^11^C-DAG spots located in the associative areas distant from the lesion between 2 weeks and 1 month after injury; one of the possible features of neural recovery in the intact brain related to PI metabolism in PET imaging [102].

In a rat model, ^11^C-DAG has also been used for assessing the myocardial PI turnover [103]. ^11^C-DAG incorporation into intermediate PI metabolites was seen both in the infarcted and the non-infarcted myocardium at 7 days after myocardial infarction. Furthermore, ^11^C-DAG has been used to evaluate the PI-mediated angiotensin II signalling pathway. In a rat model of infarcted myocardium, ^11^C-DAG assessed the effect of angiotensin-converting enzyme inhibitor (i.e. captropril) on activated PI metabolism in order to reduce left ventricular remodelling in the early phase of myocardial infarction [104]. After 3 weeks of treatment with captropril, a significant decrease of ^11^C-DAG uptake was seen in the infarcted myocardium compared to the non-infarcted myocardium, while ^201^Tl imaging showed decreased uptake in the infarcted myocardium with and without captopril treatment. In patients with myocardial infarction, ^11^C-DAG has been used to evaluate the PI myocardial turnover and the left ventricular remodelling [105]. In 13 patients with myocardial infarction, ^11^C-DAG was significantly correlated with the myocardium-to-left atrium chamber ratio, the left ventricle ejection fraction, and the brain natriuretic peptide concentration. Significant increase of ^11^C-DAG uptake was seen in the remote viable region of myocardial infarction patients compared to healthy subjects. DAG has also been labelled with ^18^F to trace the PI metabolism into the ventricular myocardium [106]. ^18^F has a longer half-life, and ^18^F-DAG may be more useful for clinical routine than ^11^C-DAG. In the rat myocardium, 3, 5, 10, and 30 min after i.v. injection of 1-[4-[18F]fluorobutyryl]-2-palmitoylglycerol (C4, C16), ***^18^F-DAG ***reflected the myocardium PI metabolism in the early phase (3 to 5 min); in the late phase (≥ 30 min) after i.v. injection,^18^F-DAG may also reflect the PE metabolism. Quantification of PI metabolism requires further optimization with a ^18^F-radiolabelled DAG compound closer to the PI metabolism than ^18^F-DAG (C4, C16) [106]. Table 4 summarises the PI-based radiolabelled molecular imaging probes.

**Table 4:** Phosphatidylinositol-based radiolabelled molecular imaging probes

Molecular target	Molecular probes	Biological functions	Isotopes	Imaging techniques
PI	inositol ^a^	PI brain metabolism	^11^C	PET(CT)
PI	DAG	PI myocardial turnover	^11^C, ^18^F	PET(CT)
PI	DAG	PI neuronal turnover	^11^C	PET(CT)

^**a **^intact inositol: no acetylated analogues. DAG, diacylglycerol; PET(CT), positron emission tomography (computed tomography); PI, phosphatidylinositol.

### SM target

SM is a cholinophospholipid located in the outer leaflet of the membrane bilayer [19]. Liposomes composed of SM have been experimentally used for tumour imaging. In a mouse tumour model, serum stable ***SM liposomes-***encapsulating ^67^Ga prepared in a lipid phase with cholesterol (SM/cholesterol molar ratio, 2:1) enhanced blood circulation and increased ^67^Ga delivery to the tumour [107]. Tumour-to-blood activity ratio (*T*/*B*) and tumour index (TI = *T*/*B *× percentage dose per gram) were higher at 24, 48 and 72 h post-injection.

Neutral SM liposomes with encapsulated ^67^Ga in an aqueous phase offer better radiolabelling properties and ^67^Ga accumulation for tumour imaging [108]. Table 5 summarises the SM-based radiolabelled molecular imaging probes.

**Table 5:** Sphyngomyelin-based radiolabelled molecular imaging probes

Molecular target	Molecular probes	Biological functions	Isotopes	Imaging techniques
SM	liposomes ^a^	tumour imaging	^67^Ga	SPECT(CT)

^a ^nanocarrier SM liposomes encapsulating ^67^Ga in lipid phase or aqueous phase. SM, sphyngomyelin; SPECT(CT), single-photon emission computed tomography (computed tomography).

## Conclusion

In nuclear medicine, the transbilayer phospholipid targets are used in preclinical and clinical research for SPECT-CT and PET-CT molecular imaging with radiolabelled probes to assess key biological functions:

1. the *PS target *allows the visualisation, characterisation, and measurement of apoptosis or necrosis, and thrombosis with radiolabelled annexin V and the ^99m^Tc or ^18^F-labelled C2A domain of synaptotagmin I and the ^99m^Tc-labelled HYNIC-lactadherin; ^99m^Tc-labelled synthetic PSBP-6-SAAC probe has been designed for molecular imaging of cell death. PS targeting probes may allow imaging of tumour endothelium vasculature with ^74^As-bavituximab. ^77/76^As-bavituximab may also serve for β- radioimmunotherapy of tumours with PS exposed endothelium tumour vasculature.

2. the *PE target *allows imaging of apoptosis or necrosis with ^99m^Tc-HYNIC-duramycin. It may also allow tumour imaging with radiolabelled ethanolamine and N,N' dimethylethanolamine.

3. the *PS and PE targets *allow imaging of necrosis with the radiolabelled ^64^Cu-bis-DOTA hypericin.

4. the *PC target *allows assessment of choline metabolism with ^11^C-choline or ^18^F-fluorocholine, and also the evaluation of the acetate and acetoacetate metabolism with ^11^C-acetate or ^18^F-fluoroacetate and ^11^C-acetoacetate.

5. the *PI target *allows evaluation of the neuronal and myocardial PI turnover with ^11^C or ^18^F-labelled DAG, and also with ^11^C-inositol.

6. the *SM target *may be used with liposomes encapsulating ^67^Ga for imaging purposes.

7. Translation of preclinical research to clinical research will be necessary to optimally assess the pharmacokinetics of radiolabelled probes for molecular imaging of transbilayer phospholipids.

## Abbreviations

AA: amino acids; ATP: adenosine triphosphate; CT: computed tomography; C2A-GST: C2A Synaptotagmin domain I glutathione-S-transferase; CA4P: combretastatin A4 phosphate; DAG: diacylglycerol; DOTA: 1,4,7,10-tetraazacyclododecane-N, N', N″, N″'-tetraacetic acid; ECM: external leaflet of the cell membrane; FITC: fluorescein isothiocyanate; ^18^F-FCH: ^18^F-fluorocholine; ^18^F-FDG: ^18^F-fluorodeoxyglucose; kDa: kilodalton; HYNIC: hydrazinonicotinamide; i.e.: id est (in example with complete enumeration); e.g.: exampli gratia (for example with incomplete enumeration); ICM: internal leaflet of the cell membrane; M: molar; 10^-9 ^M: nanomolar; MI: molecular imaging; MRI: magnetic resonance imaging; MW: molecular weight; NSCLC: non-small cell lung cancer; PA: phosphatidic acid; PC: phosphatidylcholine; PE: phosphatidylethanolamine; PET-CT: positron emission tomography-computed tomography; p.i: post-intra-venous injection; PI: phosphatidylinositol; PIP: phosphatidylinositol 4-phosphate; PIP 2: phosphatidylinositol 4,5-diphosphate; PIP 3: phosphatidylinositol 3,4,5-triphosphate; PS: phosphatidylserine; SAAC: single amino acid chelator; SM: sphyngomyelin; SPECT-CT: single-photon emission computed tomography-computed tomography; TRAIL: tumour necrosis factor-related apoptosis-inducing ligand; TUNEL: terminal deoxynucleotidyl transferase-mediated dUTP nick end labelling.

## Competing interests

The authors declare that they have no competing interests.

## Authors' contributions

TB has made substantial contribution in drafting the manuscript. FP has made substantial contribution in reviewing the manuscript. All authors read and approved the final manuscript.

## Authors' information

TB is nuclear medicine physician (MD, PhD) and Adjunct Professor at The University of Western Ontario in the department of Medical Imaging (London, ON, Canada).

FP is imaging program leader (PhD), Assistant Scientific Director at The Lawson Health Research Institute (LHRI), Professor at The University of Western Ontario in the departments of Medical Imaging, Medical BioPhysics and Physics, and Chief Medical Physicist at St. Joseph's Health Care and London Health Sciences Centre (London, ON, Canada).
